# Molecular identification and functional characterization of a cyanogenic glucosyltransferase from flax (*Linum unsitatissimum*)

**DOI:** 10.1371/journal.pone.0227840

**Published:** 2020-02-05

**Authors:** Michael Kazachkov, Qiang Li, Wenyun Shen, Liping Wang, Peng Gao, Daoquan Xiang, Raju Datla, Jitao Zou

**Affiliations:** 1 National Research Council Canada, Saskatoon, Saskatchewan, Canada; 2 Department of Plant Science, University of Saskatchewan, Saskatoon, Saskatchewan, Canada; Brigham Young University, UNITED STATES

## Abstract

Flax seed has become consumers’ choice for not only polyunsaturated alpha-linolenic fatty acid but also nutraceuticals such as lignans and soluble fiber. There is, however, a major drawback of flax as a source of functional food since the seeds contain significant level of cyanogenic glucosides. The final step of cyanogenic glucoside biosynthesis is mediated by UDP-glucose dependent glucosyltransferase. To date, no flax cyanogenic glucosyl transferase genes have been reported with verified biochemical functionality. Here we present a study on the identification and enzymatic characterization of a first flax cyanogenic glucosyltransferase, LuCGT1. We show that LuCGT1 was highly active towards both aliphatic and aromatic substrates. The *LuCGT1* gene is expressed in leaf tissues as well as in developing seeds, and its expression level was drastically reduced in flax mutant lines low in cyanogenic glucosides. Identification of LuCGT1 provides a molecular handle for developing gene specific markers for targeted breeding of low cyanogenic glucosides in flax.

## Introduction

Cyanogenic glucosides are found in plant species of more than 130 families and have important functions in plant defense against herbivores and pathogens [1–3]. Unfortunately as constituents in plant-based food cyanogenic glucosides are extremely undesirable because they can be converted to hydrocyanic acid, a highly poisonous compound, when encounter glycosidase or under low pH in the digestive tracks of animals [4–10]. Flax (*Linum unsitatissimum*) is one of the major economic crops known to possess high level of cyanogenic glucosides, linamarin and lotaustralin [11]. Flax seed is a valuable source of functional food enriched with polyunsaturated alpha-linolenic fatty acid and soluble fiber. Owing to concerns of adversary effects of cyanogenic glycosides, it is generally advised that dietary consumption of flax seed is to be restricted to a limited amount. Flax seed is also one of the richest sources of lignans for the nutraceutical supplements industry. Unfortunately, structural and physical similarities render extraction of lignans free of cyanogenic compounds from flax seed extremely challenging. Hence, a major goal of flax quality improvement is the reduction of cyanogenic glucosides in seed.

The biosynthetic pathway for cyanogenic glucosides has been extensively studied in different plants including flax (*Linum unsitatissimum)* [11–13], cassava [14–17], sorghum [6, 9, 10, 14, 17–22], *Trifolium repens* [23], *Prunus serotina* [7–8]. Existing literature [12, 24] established that the last step in the biosynthetic pathway of cyanogenic glucosides involves glycosyltransferase using UDP-glucose as glucosyl donor. The glucosyltransferase reaction attaches a sugar moiety to a cyanogenic glucosyl acceptor through a glucosidic bond. It is a biochemical step frequently found in secondary metabolism and serves to stabilize and store the metabolites that otherwise would be unstable or toxic to the cell. Genome based bioinformatics analysis showed that there are over one hundred putative glucosyltransferase genes in the genome of flax [25]. Glucosyltransferases, despite of well characterized function domains, are known to be promiscuous in terms of substrate specificity. To date, no genes encoding for cyanogenic glucosyltransferase from flax have been reported. Herein we report the molecular and biochemical characterization of the first flax cyanogenic glucosyltransferase, LuCGT1. Identification of this gene provides a molecular handle for breeding efforts to reduce cyanogenic glucoside level through either molecular marker-assisted selection or targeted gene-knockout endeavors.

## Materials and methods

### Plant materials and growth conditions

Flax cultivar CDC Bethune (wild type) was grown in growth chambers with 8 hour dark / 16 hour illumination (75–100 μE m^-2^ s^-1^). The day/night temperature was maintained at 22/17°C. All biochemical analyses were performed with leaves harvested from one month old plants except developing seeds collected 6 or 17 days after flowering. The three EMS flax mutant lines low in cyanogenic glucocides [26], UGG102-2, UGG146-1 and Double Low (DL), kindly provided by Dr. Scott Duguid, Morden Research Station, Agriculture and Agrifood Canada, were raised at identical conditions as that for CDC Bethune.

### Strains and reagents

Yeast strains: BY4741 (WT, MATa hisΔ leuΔ metΔ uraΔ) were purchased from European *Saccharomyces cerevisiae* archive for functional analysis (EUROSCARF). ^14^C-Uridine 5′-diphosphoglucose ^14^C-UDP-glucose was purchased from American Radiolabeled Chemicals Inc. Substrates and other chemicals used in this study were obtained from Sigma-Aldrich. Media and plates were prepared according to Invitrogen’s recipe described for the pYES2.1 TOPO TA Cloning Kit.

### PCR amplification and construct generation

The full-length cDNA sequence of candidate glucosyltransferase genes were derived from a flax developing seed cDNA library as previously described [27]. For full length cDNA cloning, the complete coding regions of UGT85Q1, UGT74S1 and UGT84G3 were amplified with the following primers incorporating *Sph*I and *Bam*HI sites: UGT85Q1-F2/ UGT85Q1-R2, UGT74S1-F2/ UGT74S1-R2 and UGT84G3-F2/ UGT84G3-R2. The primers are listed in S1 Table. The purified fragments were digested with *Sph*I and *Bam*HI for UGT85Q1, and *Sph*I and *Bgl*II for UGT74S1 and UGT84G3, respectively. The digested fragments were then ligated to the pQE70 vector for sequencing versification. For the yeast expression system, the coding regions of UGT85Q1, UGT74S1 and UGT84G3 were amplified with UGT85Q1-F1/ UGT85Q1-R1, UGT74S1-F1/ UGT74S1-R1 and UGT84G3-F1/ UGT84G3-R1, respectively. The obtained fragments were ligated directly to the pYES2.1 vector according to manufacturer’s instruction (Invitrogen). The resulting plasmids were verified through DNA sequencing and subsequently introduced into yeast strain BY4741. To examine the promoter and coding region of UGT85Q1, five consecutive fragments were amplified with the following primers covering the whole region: Pro-F1/ Pro-R1, Pro-F2/ Pro-R2, Pro-F3/ Pro-R3, Pro-F4/ Pro-R4 and Pro-F5/ Pro-R5 (S1 Table).

### Heterologous expression in yeast

All procedures were carried out at 4°C except where indicated. Yeast strains were first grown (28°C) in 15 mL of SC-Ura medium containing 2% glucose. Protein expression induction was carried out as described in the manufacturer manual (Invitrogen). After 24 h of growth (28°C) in SC + 2% galactose + 1% raffinose, the cells were washed sequentially with distilled water and homogenization buffer (50 mM Tris–HCl, 1 mM EDTA, 0.6 M sorbitol, pH 7.4, 1 mM dithiothreitol (DTT). After centrifugation at 4,000 rpm (Eppendorf 5145C), the cells were resuspended in 1 mL homogenization buffer containing 10 μL yeast protease cocktail (Sigma), and shaken vigorously (1 min X 2 times) in the presence of acid-washed glass beads (diameter 0.5 mm). The resultant homogenate was centrifuged at 12,000 rpm for 10 min. The decanted supernatant was further centrifuged at 100,000 g for 90–120 min. The supernatant was carefully separated from the pellet and collected for further experiments. The pellet was resuspended in homogenization buffer containing 20% glycerol and frozen at -80 ^0^C along with supernatant until use. Protein concentration was measured using a Bio-Rad Protein Assay Kit for final enzyme activity calculation.

### In vitro assay of glucosyltransferase activity

Glucosyltransferase kinetic constants and substrate specificity were both determined by measuring incorporation of ^14^C-UDP-glucose incorporation into cyanogenic glycosides. Glycosyltransferase substrate specificity assessment was performed in 0.1 mL HEPES (pH 8.0, 0.1 M) buffer containing 100 μg of protein, 5mM of ^14^C-UDP glucose (0.25 nCi/nmol) substrates and 100 mM aglycones. All assays were performed at least twice. Reaction was allowed to proceed for 30 min at 30°C with 700 rpm shaking and stopped by adding 20 μL acidic acid (20%). All reactions were linear at least in 1 h range. For determination of apparent *Km* and *Vmax*, 100 μg of protein was used in the same reaction system with different concentrations of ^14^C-UDP-glucose and Acetone Cyanohydrin. The reaction products were spotted on Merck silica G60 TLC plates and separated in Ethyl Acetate : Acetone : Dichloromethane : Methanol : Water (40 : 30 : 12 : 10 : 8) system. Spots corresponding to different cyanogenic glucoside products were scraped off and 14°C incorporation was scintillation counted. Calculation of concentration acetone cyanohydrin (undissociated) in equilibrium has been performed in accordance with a previous study [20].

### Total RNA extraction and qRT-PCR

Isolation and purification of total RNA for quantitative RT-PCR (qRT-PCR) was performed using mini plant RNA extraction Kit according to the manufacturer’s introductions (QIAGEN). Approximately 1 μg of total RNA was used for cDNA synthesis using QuantiTect reverse transcription kit (QIAGEN). The obtained cDNA was diluted fifty times to conduct qRT-PCR. For each reaction, a volume of 15 μL containing 5 μL of the diluted cDNA, 7.5 μL of SYBR Green Master Mix (Bio-Rad) and 0.6 μL of primer (5 μM). The reference gene *Actin* and the target gene *UGT85Q1* were amplified using primers Actin-F/Actin-R and UGT85Q1-F3/ UGT85Q1-R3, respectively (S1 Table).

## Results

### Identification of flax cyanogenic glucosyltransferase

We were specifically interested in cyanogenic glucosyltransferases that are highly expressed in developing seeds. Previous reports using crude enzyme preparations from flax demonstrated that the flax glucosyltransferase possesses properties resembling that of enzyme from sorghum [28]. We thus used the deduced amino acid sequence of the *sorghum bicolor* cyanogenic glucosyltrasferase [19, 22] as a reference to conduct BLAST search against a flax EST database generated from developing seed [27]. This resulted in the identification of 38 EST entries that displayed significant sequence homology to the sorghum enzyme (S1 Fig).

Three candidate genes, previously annotated as UGT85Q1 (GenBank accession: ADV36300) [25], UGT74S1 (Genbank: JX011632.1) and UGT84G3 (GenBank: AFJ52991.1) were found to be abundantly represented in the developing seed EST database [27]. Full length sequence of the cDNA was established based on sequencing of multiple independent cDNA clones. UGT74S1 was previously shown to possess UDP-glucosyltransferase activity towards secoisolariciresinol and contribute to lignan biosynthesis in flax [29]. The function of UGT84G3 is currently unknown. This study focuses on UGT85Q1, which is herein designated as *LuCGT1* and encodes a polypeptide of 492 amino acids. The deduced amino acid sequence of LuCGT1 contains at its C-terminal region a putative nucleotide-diphosphate-sugar binding domain, ^369^WCPQEDVLNHPAVGGFLTHCGWGSIIESLTAGVPLLCWPFFGDQ^412^, commonly known as the “plant Secondary Product Glycosyltransferase” (PSPG) [30]. There are two other close related homologs in flax, UGT85Q3 (AFJ53000) and UGT85Q2 (AFJ52999), to which LuCGT1 displays 69% and 66% sequence identity, respectively.

### LuCGT1 encodes a functional cyanogenic glucosyltransferase

**Fig 1:**
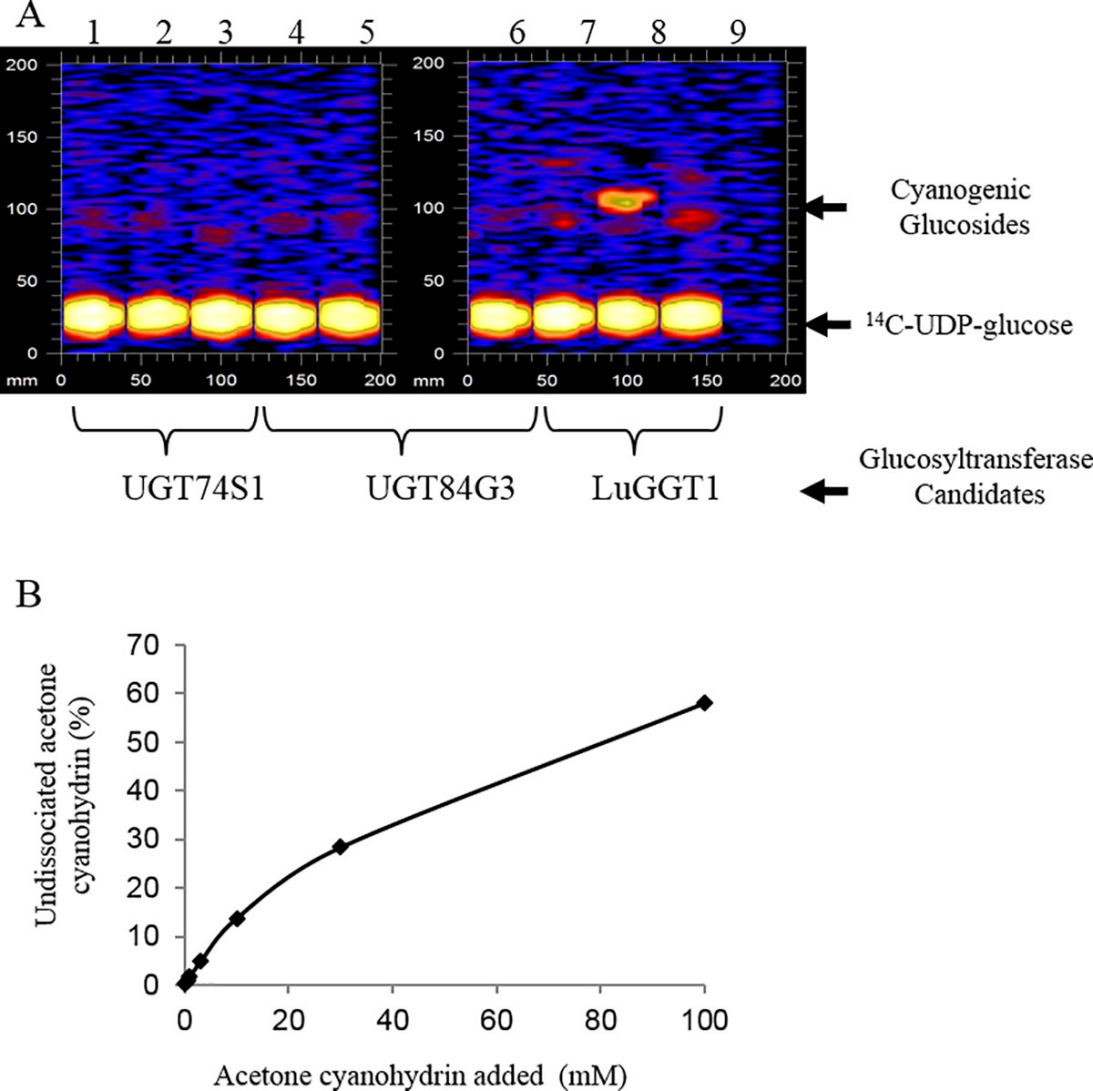
Glucosyltransferase activity of LuCGT1 expressed in yeast. (A) Cyanohydrin UDPG-glycosyltransferase assay using ^14^C-UDP-glucose in the presence of madelonitrile (lane 1, 4, 7), acetone cyanohydrin (lane 2, 5, 8), and negative control without any glucosyl acceptor (lane 3, 6, 9). Formation of radiolabeled product is evident in yeast strain expressing LuCGT1 (lane 8). (B) Concentration of acetone cyanohydrin in equilibrium at cyanogenic glucosyltransferase assay. Data for the amount of undissociated acetone cyanohydrin were calculated according to Mederacke et al [15].

We inserted the full length cDNA of *LuCGT1*, *UGT74S1*, and *UGT84G3* into the yeast expression vector pYES2.1. The yeast expression constructs were subsequently introduced into yeast strain BY4741. We conducted enzymatic assays using homogenate of yeast cultures upon galactose induction. Our initial assays employed acetone cyanohydrin and mandelonitrile as glucosyl acceptors and ^14^C-UDP-glucose as glucosyl donor. Fig 1 illustrates one representative TLC plate from our experiments. Repeated assays using lysate of BY4741 harboring *UGT74S1* or *UGT84G3* detected no appreciable enzyme activity with either glucosyl acceptors. These results showed that the yeast expression system possessed no background cyanogenic glucosyltransferase assays, and that despite of high sequence homology, neither *UGT74S1* nor *UGT84G3* encoded the enzyme of our interest. Yeast lysate harboring *LuCGT1*, on the other hand, displayed strong activity towards acetone cyanohydrin. It should be noted, however, assays with the same lysate failed to detect activity when mandelonitrile was used as glucosyl acceptor (Fig 1A, lane 8), indicating an apparent substrate preference of this enzyme. The undissociated acetone cyanohydrin concentration in the *in vitro* enzymatic assay of LuCGT1, determined according to Mederacke et al [18], was found to be less than 0.2% in *Km* range of concentration (Fig 1B).

### Enzyme kinetics of LuCGT1

**Fig 2:**
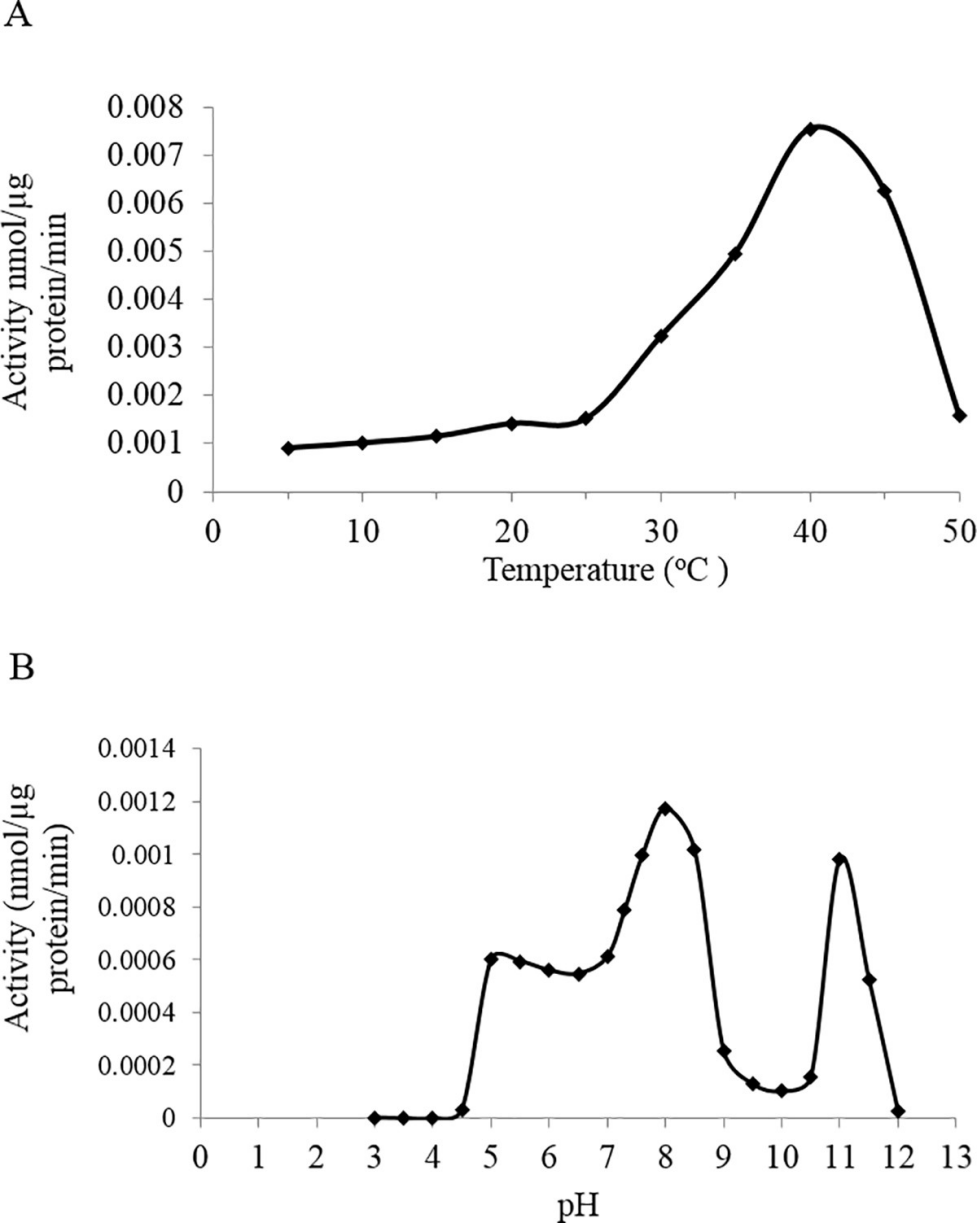
Enzyme kinetics of LuCGT1. (A) Temperature dependence of LuCGT1. Activity of cyanogenic glucosyltransferase was determined by the standard assay, modified by varying temperature. (B) pH dependence of LuCGT1. Activity of cyanogenic glucosyltransferase was determined by standard assay conditions as described in “Materials and methods”, modified by varying pH.

We further studied the properties of LuCGT1 by assessing the optimal biochemical parameter of the glucosyltransferase reaction. Temperature ranging 5 to 50°C, and pH from 3 to 12 were tested for optimal temperature and pH. Temperature optimum for LuCGT1 was found at 40–42°C (Fig 2A), and the pH optimum for this cyanogenic glucosyltransferase was observed at between pH 7.5 and 8.5 (Fig 2B).

After optimization of the enzymatic assay and determination of the reaction pH and temperature optimum, we were able to assess the apparent *Km* and *Vmax* of LuCGT1 for acetone cyanohydrin and UDP-Glucose. The apparent K_m_ for acetone cyanohydrin and uridine diphosphoglucose were at 61.3 ± 4.5 μM and 248 ± 56 μM, respectively (Table 1). The four folds difference in *Km* between acetone cyanohydrin and UGP-Glucose were consistent with the known physical concentration of these substrates in plant tissues and the fact that the acetone cyanohydrin itself is very unstable, particularly under low pH conditions. Under the optimal pH and at 2x *K*_*m*_ concentration, it is estimated that there would be only about 0.2 percent of acetone cyanohydrin that remains in undissociated form suitable for enzyme action.

**Table 1:** The apparent *Km* and *Vmax* of LuCGT1 towards acetone cyanohydrin and UDP-glucose.

Substance	Apparent *K*_*m*_	Apparent *V*_*max*_
Acetone Cyanohydrin	61.3 ± 4.5 μM	0.382 ± 0.021 nmol/min
Uridine diphosphoglucose	248 ± 56 μM	0.42 ± 0.052 nmol/min

### Substrate specificity of LuCGT1

Our initial enzyme activity assessment using acetone cyanohydrin and mandelonitrile indicated a glucosyl acceptor substrate selectivity of LuCGT1. Given that cyanogenic glucosyltransferases often display broad substrate specificity during in vitro assays, we next conducted substrate preference assays with a broader range of potential glucosyl acceptors. High level of glucosyltransferase activity was detected with 3-hydroxypropionitrile, lactonitrile, glyconitrile and 3-hydroxybutyronitrile glycosylation (S2 Fig). The LuCGT1 also displayed significant activity towards benzyl alcohol. The spots on the TLC plate corresponding to glucoside products were scraped off, and specific activities with each glucosyl acceptor relative to that of acetone cyanohydrin (referred as 100%) were assessed. Data presented in Table 2 indicated that this newly discovered flax glucosyltransferase had a broad substrate specificity, which was consistent with previous studies on similar enzymes from other plant species [13, 19]. Due to a lack of a commercial source, we were unable to investigate if LuGCT1 possessed activity towards 2-methybutyronitrle, which was known as the precursor for lotaustralin.

**Table 2:** Broad substrate specificity of LuCGT1.

Substrate	Activity
salicylic acid	Non detected
geraniol	Trace
glyconitrile	58% ± 1.8
3-hydroxypropionitrile	69.2% ± 2.4
3-hydroxybutyronitrile	57.3% ± 14.4
benzyl alcohol	30.53% ± 10
2-hydroxybutyronitrile	46.64% ± 6.2
lactonitrile	59.5% ± 4.4
mandelonitrile	Non detected
acetone cyanohydrin	100%

### Expression profile of LuCGT1

**Fig 3:**
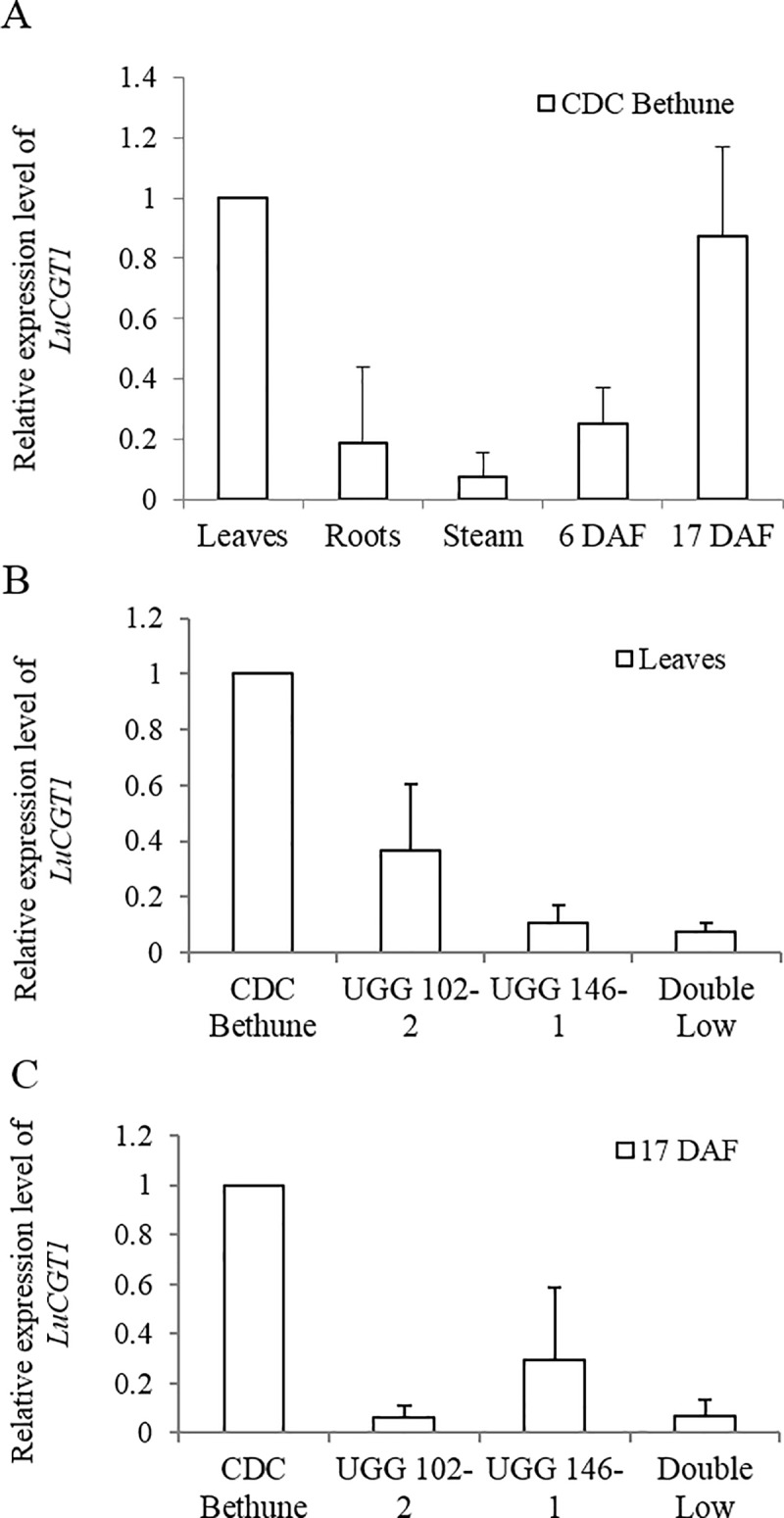
Tissue expression analysis of *LuCGT1* using real-time quantitative RT-PCR. (A) *LuCGT1* expression profile in flax cultivar Bethune; (B) relative expression level of *LuCGT1* in leaf tissues of low cyanogenic glucoside lines; (C) relative expression level of *LuCGT1* in developing seed tissues of low cyanogenic glucoside lines. For compression, the expression level of *LuCGT1* from CDC Bethune leaves or seeds was normalized to 1 using StepOne software 2.0 (Applied Biosystems). The values represent the average of three independent biological replicates. Each of biological repeat contains for technical replicates. UGG102-2, UGG 146–1, Double Low are low cyanogenic flax lines.

To investigate the expression patterns of *LuCGT1* in different tissues of *Linum unsitatissimum* plants, quantitative real-time PCR (qRT-PCR) was performed using RNA prepared from 4-week old, roots, stems, leaves, as well as developing seeds from cultivar CDC Bethune at 6 DAF (days after flowering) and 17 DAF (Fig 3A). The *LuCGT1* gene was expressed in all tissues Fexamined. But the highest expression level was detected in leaves and 17 DAF developing seeds. These results were consistent with our findings that EST corresponding to *LuCGT1* was highly abundant in the developing seed and seed coat cDNA libraries [27].

Three flax mutants, UGG 102–2, UGG146-1 and Double Low, have been known to possess low level of cyanogenic glucosides [26]. Two major QTLs governing cyanogenic glucoside contents were mapped to two linkage groups localized on Chromosome 1 and Chromosome 6/13 [26]. LuCGT1 is locates on chromosome Lu10 (12,164,270 bp to 12,165,855 bp) based on latest released *Linum usitatissimum* reference genome (ASM22429v2, GenBank assembly [GCA_000224295.2]). We amplified the genomic DNA sequences corresponding to the promoter region and coding sequences of *LuCGT1* through PCR and conducted sequence comparison with its parent line, cultivar CDC Bethune. No mutations were found in the LuCGT1 gene in any of the three mutants. We concluded that *LuCGT1* was not the underlying genetic lesion causing low cyanogenic glucoside content in these mutant lines. We then performed transcript level analysis on leaf tissues and developing seeds of the mutants and compared with that of CDC Bethune. Results from qRT-PCR show that in both leaf tissues (Fig 3B) and developing seed (Fig 3C). The transcript levels of *LuCGT1* were found to be significantly lower in the low cyanogenic glucoside flax mutant lines. These results suggest a correlation between LuCGT1 transcript level and the accumulation of cyanogenic glucoside in flax.

## Discussion

Flaxseed has a major quality shortcomings as a source of functional food due to the presence of cyanogenic glucosides. In the present study, we focused on the last step in the biosynthetic pathway of cyanogenic glucosides, the glucosylation of the cyanogenic compounds. A bioinformatics survey of putative UDP-glucosyltransferase genes in flax was previously reported [25]. However, since UDP-glucosyltransferases are known to have broad substrate specificities, a careful biochemical characterization is required to conclusively assign enzymatic function to candidate genes. A number of enzymes have been isolated from plant tissues which catalyze the transfer of glucose from UDP-glucose to aliphatic or aromatic hydroxyl groups. We employed the sorghum cyanogenic glucosyltransferase [19] as a reference sequence to conduct bioinformatics search against a flax seed EST database we previously developed [27]. Based on BLAST search results, we were able to select three candidate genes. When expressed heterologously in yeast, only LuCGT1 exhibited cyanogenic glucosyltransferase activity. The other two candidate flax glucosyltransferase, UGT74S1 and UGT84G3, despite of being closely related at the deduced amino acid sequence level, displayed no detectable activity under our assaying conditions. UGT74S1 was shown to be a specialized glucosyltransferase in lignan biosynthesis [29]. Our results show that LuCGT1 is also active towards cyanohydrin intermediates and is highly expressed during seed development. LuCGT1 can utilize aromatic substrates, but it was not active towards mandelonitrile, a highly active substrate for the sorghum gucosyltransferase [22]. LuCGT1 also exhibited activities towards a few other compounds belonging to completely different class. Whether such activities have physiological relevance in flax remains to be studied. The conversions of amino acid to the corresponding oximes have been shown to be associated with microsomes [21, 28, 31] through association with the membrane bound cytochrome P450s [32, 33]. However, in our experiments using yeast lysate, enzyme activity was found to be associated with the soluble fractions of the cell homogenates, which is consistent with findings from the sorghum enzyme [32, 33].

In this study we were also interested in whether there would be mutations in *LuCGT1* in the genome of three low cyanogenic glucoside flax mutant lines. We found no mutations neither in the gene-coding regions nor promoter sequences when compared with that of cultivar CDC Bethune. We cannot rule out the possibility that there are other cyanogenic glucosyltransferase isoforms in flax. However, our RT-PCR experiments designed to assess the transcript level of this gene indicated that the low cyanogenic flax lines had relatively low expression level of *LuCGT1*, in leaves as well as in developing seeds. Hence, even though *LuCGT1* was not the genetic lesion underlying the low cyanogenic glucoside trait, its expression level likely reflected a subdued overall cyanogenic glucoside biosynthesis pathway in the mutants. A notable feature of cyanogenic glucoside synthesis in plants is that of the formation of metabolons between cytochrome P450 and glucosyltransferase. In this context, LcCGT1 will be a valuable molecular tool for probing biochemical mechanisms governing metabolic channeling in flax [31, 32]. The molecular cloning of *LuCGT1* also provides a molecular guide for targeted breeding of low cyanogenic glucosides flaxseed. A low cyanogenic glucosides and/or cyanogenic glucoside-free flux cultivar will bring the full potential and total utilization of flax as a crop, and substantially simplify the extraction procedure of a number of nutraceutical compounds from flax.

## Supporting information

S1 Fig BLAST search alignment. Thirty eight flax EST entries with significant sequence homology to the sorghum enzyme were identified using sorghum glucosyltransferase.
(TIF)

S2 Fig Substrate preference of LuCGT1. Glucosyl acceptor used in the assasys were salicylic acid (lane 1), geraniol (lane 2), glyconitrile (lane 3), 3-hydroxypropionitrile (lane 4), 3-hydroxybutyronitrile (lane 5), benzyl alcohol (lane 6), 2-hydroxybutyronitrile (lane 7), lactonitrile (lane 8), acetone cyanohydrin (lane 9, and lane 11), Mandelonitrile (lane 12). Lane 10 as control without glucocyl acceptor substrates.
(TIF)

S1 Table Primers used in this study. (DOCX)
