# Supplementary figures and images for: Molecular identification and functional characterization of a cyanogenic glucosyltransferase from flax (Linum unsitatissimum)
Source: PLoS One. 2020 Feb 5;15(2):e0227840. doi: 10.1371/journal.pone.0227840 (PMC7001965; doi:10.1371/journal.pone.0227840)

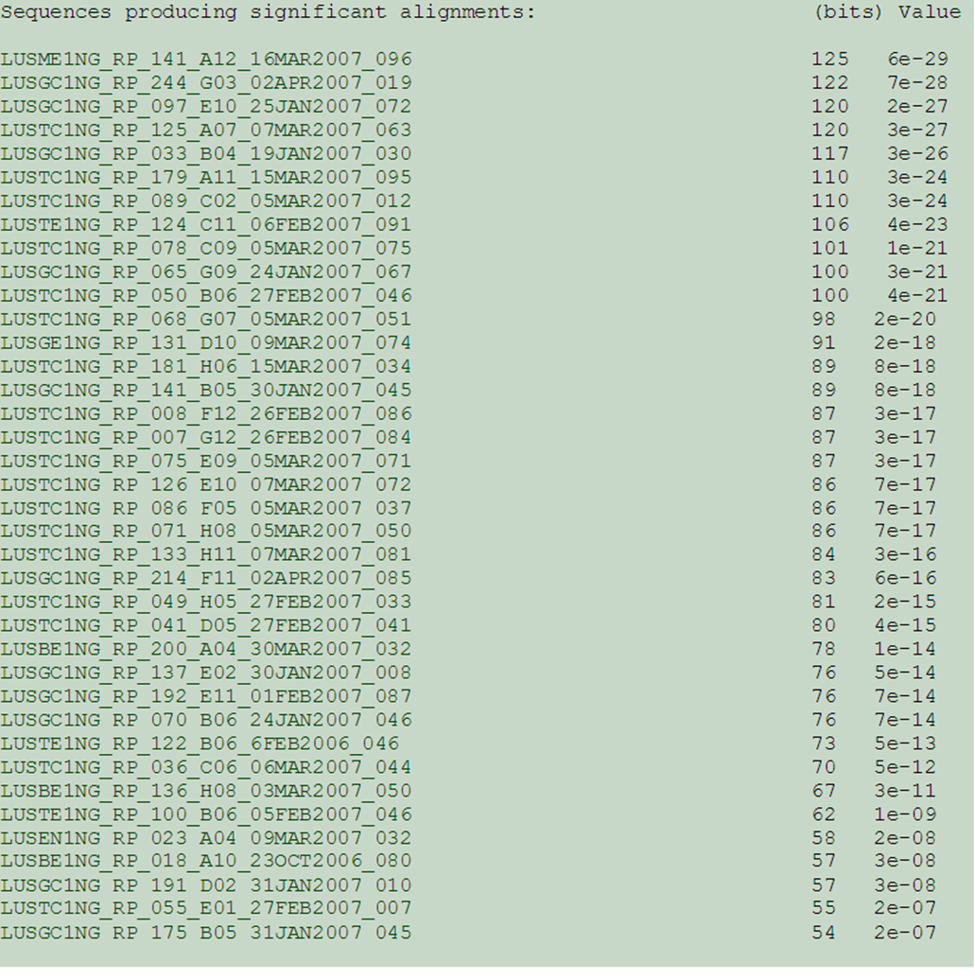

Supplement: S1 Fig — Thirty eight flax EST entries with significant sequence homology to the sorghum enzyme were identified using sorghum glucosyltransferase. (TIF) [file pone.0227840.s001.tif]

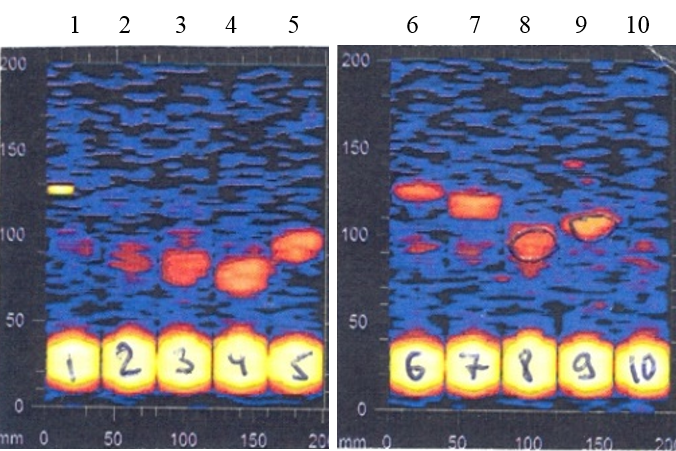

Supplement: S2 Fig — Glucosyl acceptor used in the assasys were salicylic acid (lane 1), geraniol (lane 2), glyconitrile (lane 3), 3-hydroxypropionitrile (lane 4), 3-hydroxybutyronitrile (lane 5), benzyl alcohol (lane 6), 2-hydroxybutyronitrile (lane 7), lactonitrile (lane 8), acetone cyanohydrin (lane 9, and lane 11), Mandelonitrile (lane 12). Lane 10 as control without glucocyl acceptor substrates. (TIF) [file pone.0227840.s002.tif]
