# Supplementary material for: Molecular identification and functional characterization of a cyanogenic glucosyltransferase from flax (Linum unsitatissimum)
Source: PLoS One. 2020 Feb 5;15(2):e0227840. doi: 10.1371/journal.pone.0227840 (PMC7001965; doi:10.1371/journal.pone.0227840)
Supplement: S1 Table — (DOCX) [file pone.0227840.s003.docx]

**Supplementary Table 1.** Oligonucleotide primers

| Primer | | Sequence (5’-3’) |
| --- | --- | --- |
| UGT85Q1-F2  UGT85Q1-R2  UGT74S1-F2  UGT74S1-R2  UGT84G3-F2  UGT84G3-R2  UGT85Q1-F1  UGT85Q1-R1  UGT74S1-F1  UGT74S1-R1  UGT84G3-F1  UGT84G3-R1  Pro-F1  Pro-R1  Pro-F2  Pro-R2  Pro-F3  Pro-R3  Pro-F4  Pro-R4  Pro-F5  Pro-R5  Actin-F  Actin-R  UGT85Q1-F3  UGT85Q1-R3 | GCATGCGAATGGGTTCGGAAGAGGGA  GGATCCTATTTGTTGGTTCTTTGCTG  GCATGCGAATGGCGGGCGATGAGAGA  AGATCTATCGTAAAATGCCCTTTC  GCATGCGAATGGGATCCTCCTCGTCG  AGATCTATATTGCGACGACTGTTCCT  GAAATGGGTTCGGAAGAGGGAGCTG  CTACTTGGAGTTGGGTGAAAGAAC  GATATGGCGGGCGATGAAAGAGAA  TCAGCAGCTAAATTGGAAAGTTTC  GTAATGGGATCCTCCTCGTCGGAGA  CTACAACGTCGCACCGTTCCTCTTC  GGTGGGTTCTATTATTATTTTCATC  AAAAAACTCTTCATCTCATCCATAC  GCCACATAAGTGCCATGTTATTTTG  CACTGCAACTGTAGACTGTAGTAGT  TGAACCGACTCCACTGCCTCTCCAT  GTGGAGTCAGGATTGTCGGATGGAG  TCCTCACAAATACTATAAAGAAATA  CCAGATGAAGGACGGAGGACCCACC  ATGGCAGGTGGACATGCACAGACAG  GAAAGAACTTGAGAAACCAAACGAT  TGATGGTGTTAGCCACACAGTCCCC  CCACCACTGAGCACAATGTTACCGT  TCTCTTTACTACAACTCCTCGACCA  GACTCGAAACGAGGAGTAAAAGAAC | |
